# Satisfaction of surgeons with the current state of training in minimally invasive surgery: a survey among German surgeons

**DOI:** 10.1007/s00464-023-10584-y

**Published:** 2023-12-12

**Authors:** Felix von Bechtolsheim, Alfred Schneider, Sofia Schmidt, Omar Al-Aqiqi, Olga Radulova-Mauersberger, Grit Krause-Jüttler, Jürgen Weitz, Marius Distler, Florian Oehme

**Affiliations:** 1 Department of Visceral-, Thoracic and Vascular Surgery, Faculty of Medicine and University Hospital Carl Gustav Carus, Technische Universität Dresden, Dresden, Germany; 2 Centre for Tactile Internet With Human-in-the-Loop (CeTI), Technische Universität Dresden, Dresden, Germany

**Keywords:** Minimally invasive surgery, Training, Satisfaction, Germany, Robotic surgery

## Abstract

**Background:**

Minimally invasive surgery (MIS) requires intense education and training with structured supervision and feedback. However, a standardized training structure is lacking in Germany. This nationwide survey aimed to assess the current state of minimally invasive surgery (MIS) training and factors impacting surgeons' satisfaction.

**Methods:**

Between July and October 2021, an online survey was conducted among general, abdominal, and thoracic surgeons in Germany. The survey collected data on department size, individual operative experience, availability of MIS training equipment and curricula, and individual satisfaction with training. A linear regression analysis was conducted to investigate factors influencing the surgeons’ satisfaction with the MIS training.

**Results:**

A total of 1008 surgeons participated in the survey, including residents (26.1%), fellows (14.6%), attendings (43.8%), and heads of departments (15.2%). Of the respondents, 57.4% reported having access to MIS training equipment, 29.8% and 26% had a curriculum for skills lab MIS training and intraoperative MIS training, respectively. In multivariate linear regression analysis, strongest predictors for surgeons’ satisfaction with skills lab MIS training and intraoperative training were the availability of respective training curricula (skills lab: β 12.572; p < 0.001 & intraoperative: β 16.541; p < 0.001), and equipment (β 5.246; p = 0.012 & β 4.295; p = 0.037), and experience as a first surgeon in laparoscopy (β 12.572; p < 0.001 & β 3.748; p = 0.007). Additionally, trainees and teachers differed in their satisfaction factors.

**Conclusion:**

Germany lacks standardized training curricula and sufficient access to MIS training equipment. Trainees and teachers have distinct factors influencing their satisfaction with MIS training. Standardized curricula, equipment accessibility, and surgical experience are crucial for improving surgeons’ satisfaction with training.

**Supplementary Information:**

The online version contains supplementary material available at 10.1007/s00464-023-10584-y.

The increasing need for surgical services globally may be due to a decrease in the number of surgeons available to meet an increasing need [1, 2].

However, the plight is aggravated by attrition rates from surgical residencies, which can be as high as 20% [3, 4]. Residents who dropped out of surgical residency programs gave testimony that the subordination of teaching in favor of clinical duties was a primary reason for their decision [5].

Improving the quality of surgical training is debated as one of the most auspicious ways to increase the number of surgical residents [6].

Besides the fundamental impact on the attractiveness of the surgical profession to potential applicants and those enrolled in surgical residencies, training is essential to providing excellent and consistent quality of care. This is especially important in the context of minimally invasive surgery (MIS), where the learning curve is significantly slower than that of conventional open surgery [7].

The importance of adequate training in MIS is underlined by the fact that MIS is now used for many procedures, such as bariatric and reflux surgery, appendectomies, and cholecystectomies [8]. Robot-assisted surgery adds a new aspect to MIS that has only recently begun to gain considerable momentum [9]. However, the evolution of surgical training has not kept pace with developments, and the importance of adequate training in MIS remains paramount.

Although fellowships for MIS were introduced in the United States as early as 2007, significant deficits in MIS training still exist many years later [10]. A survey by Gardner et al. found most surgical residents felt unable to perform advanced laparoscopic surgery after completing their surgical training, and their instructors observed a decline in surgical competency compared to previous cohorts. Interestingly, both groups reported that trainees were given little autonomy to perform advanced procedures [11].

This trend was anticipated as early as 2009 by Hedrick et al., who noted a shift in the learning curve of laparoscopic surgery to the later years of training, potentially harming the learning of technical skills in the early years of surgical training, whereas comparable open surgery was mastered much earlier in training [12].

To gain significant MIS operative experience, specialized training inside and outside the operation room (OR) is required, as studies have shown the beneficial effect of adequate training on intraoperative performance [13–15].

Therefore, besides the obvious beneficial effect of training on the operative performance of surgeons, an adequate MIS education and training concept could attract new surgeons and retain residents within the residency programs. This nationwide survey aims to obtain an up-to-date overview of the MIS training reality in Germany, with a special focus on the satisfaction level with the training situation and corresponding influencing factors.

## Materials and methods

**Fig. 1:**
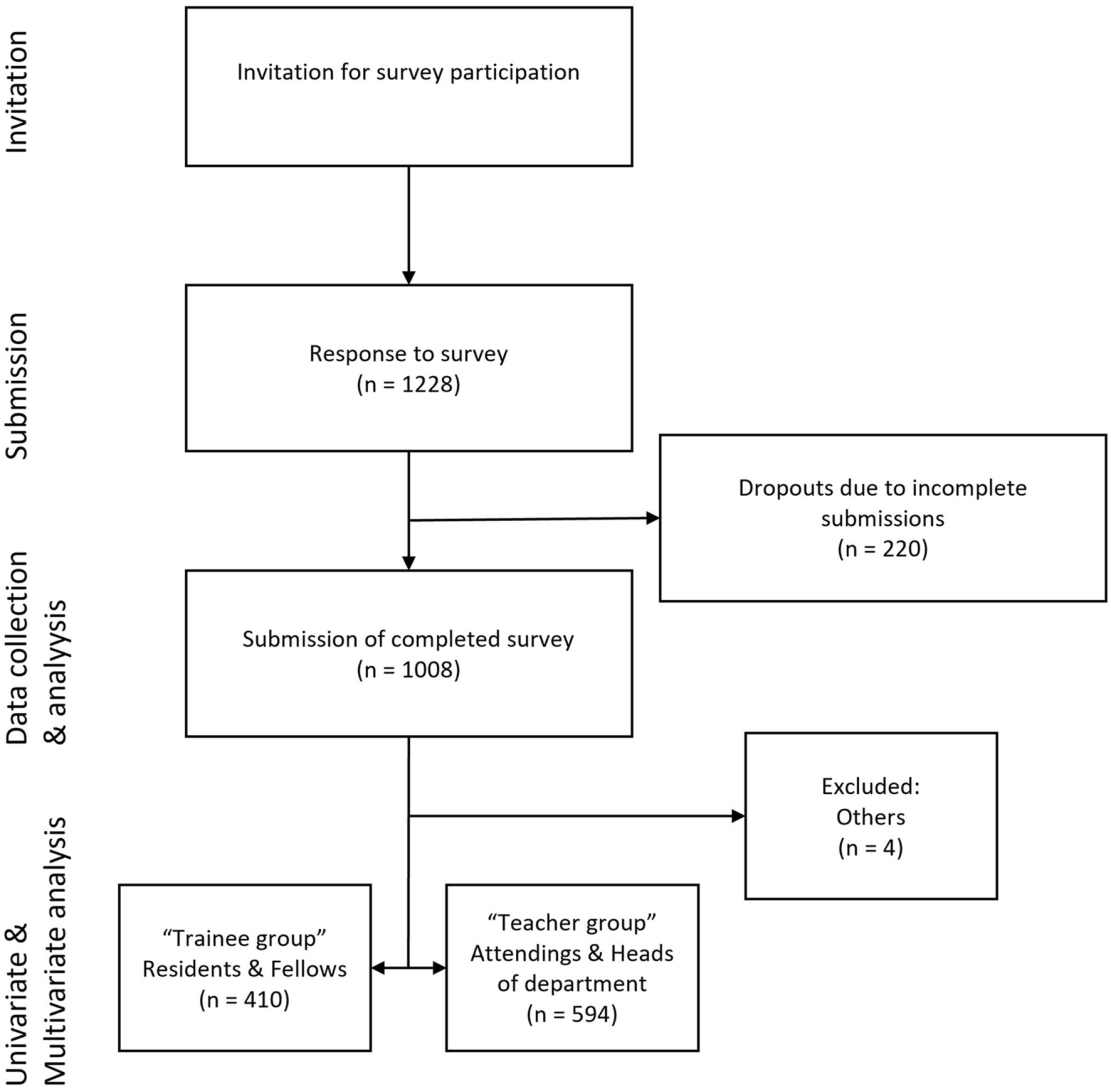
Trial scheme

This trial was conducted as an online survey through LimeSurvey© (Fig. 1) and adheres to the Checklist for Reporting Results of Internet E-Surveys (CHERRIES) guideline [16]. For recruitment of participants emails were sent to all publicly accessible email addresses of general, abdominal and thoracic surgeons and general surgical departments in Germany. The call for participation was repeated four times via the same email distribution list at regular intervals over a four-month period between July and October 2021. Participation in the survey was closed in November 2021. The survey was approved by the ethics committee of the Technische Universität Dresden (approval number: BO-EK-59012021).

### Survey

The survey comprised up to 35 questions in German. At the beginning of the survey, all participants were informed that their participation was voluntary and anonymous. The questions were arranged into three thematic subgroups:

1. Questions on personal information, such as years of practice, specialization, and surgical experience for laparoscopic, thoracoscopic, and robot-assisted procedures. Response categories were “0”, “1–25”, “26–50”, “51–100”, and “ > 100 procedures.” In addition, the survey asked about the hierarchical status. Since this hierarchy system is a special feature of the German healthcare system, the response options are defined as follows:
  - *Resident* resident surgeon in training without board certification
  - *Fellow* surgeon with board certification working under supervision
  - *Attending surgeon* surgeon with board certification supervising residents and fellows
  - *Head of department* senior surgeon supervising a surgical department
2. Questions about the employing hospital or department, such as certification as an MIS center, size (measured by the number of patient beds and surgeons), and surgical capacity (measured by the number of all minimally invasive elective, robot-assisted, oncological, and emergency surgical procedures performed in one week).
3. Questions about the MIS training content and its implementation. We distinguished between a practical skills training curriculum in dry- or wet-lab conditions (outside the OR) and an intraoperative training curriculum during operations. We also queried the provision of teaching equipment, as well as practice times. Finally, participants were asked to rate their satisfaction with their respective training situation on a scale from 0 (not satisfied) to 100 (very satisfied). This included satisfaction levels for training both inside and outside the OR, as well as the provided time and training equipment.

### Statistical analysis

The analysis included only fully completed survey data sets, resulting in no missing data. Statistical analysis was conducted using IBM SPSS Statistics version 28 (IBM Corp., Armonk, NY, USA). Continuous data normality was assessed using frequency distributions and the Kolmogorov–Smirnov test. Participant characteristics were presented as frequency distributions or median with interquartile ranges (IQRs) for continuous variables. A linear regression model was used to investigate factors influencing surgeons’ satisfaction, with significant variables from univariate analysis included in the multivariate analysis. A p-value of < 0.05 was considered statistically significant.

## Results

### Participants characteristics

The online survey included 1228 participants, of whom 1008 completed the survey while 220 dropped out (Table 1). The participants represented various hierarchy groups within the surgical profession, including residents (n = 263; 26.1%), fellows (n = 147; 14.6%), attending surgeons (n = 441; 43.8%), heads of departments (n = 153; 15.2%), and others (n = 4; 0.4%). The median length of time participants had worked in the surgical profession was 13 years (IQR 6–22).

**Table 1:** Participant and department characteristics

	n (%)	Median (25–75 Perc.)
Surgical profession		
Abdominal surgery	838 (83.1)	
General surgery	112 (11.1)	
Thoracic surgery	35 (3.5)	
Other	23 (2.3)	
Hierarchy group		
Resident	263 (26.1)	
Fellow	147 (14.6)	
Attending surgeons	441 (43.8)	
Heads of department	153 (15.2)	
Other	4 (0.4)	
Years of practice department		
Number of beds	1003 (99.5)	45 (30-60)
Number of surgeons employed	1008 (100)	14 (9–22)
Number of surgeons performing MIS regularly (min. 1/week)	1008 (100)	7.5 (5–10)
Certified center for MIS	241 (23.9)	
Minimally invasive procedures per week		
All elective surgery	1008 (100)	15 (10–20)
Robotic surgery	353 (35)	3 (2–4)
Oncological resections	938 (93.1)	3 (2–5)
Emergency surgery	995 (98.7)	5 (4–9)

The departments where participants had worked had a median of 14 (IQR 9–22) surgeons responsible for 45 (IQR 30–60) patient beds. Only 7.5 (IQR 5–10) surgeons per department performed MIS at least once a week. Additionally, 241 participants (23.9%) reported working at a center for MIS certified by a German surgical association. All participants (100%) reported to use MIS at their department, 995 participants (98.7%) used MIS for emergency surgery, 938 (93.1%) used MIS for oncological resections and 353 participants (35%) integrated robotic surgery at their department. Participants estimated to perform 15 (IQR 10–20) elective and five (IQR 4–8) MIS emergency procedures per week in their respective department. Among elective surgeries, three (IQR 2–5) procedures were oncological resections, and 3 procedures (IQR 2–4) were performed robotically assisted.

### Surgical experience

**Fig. 2:**
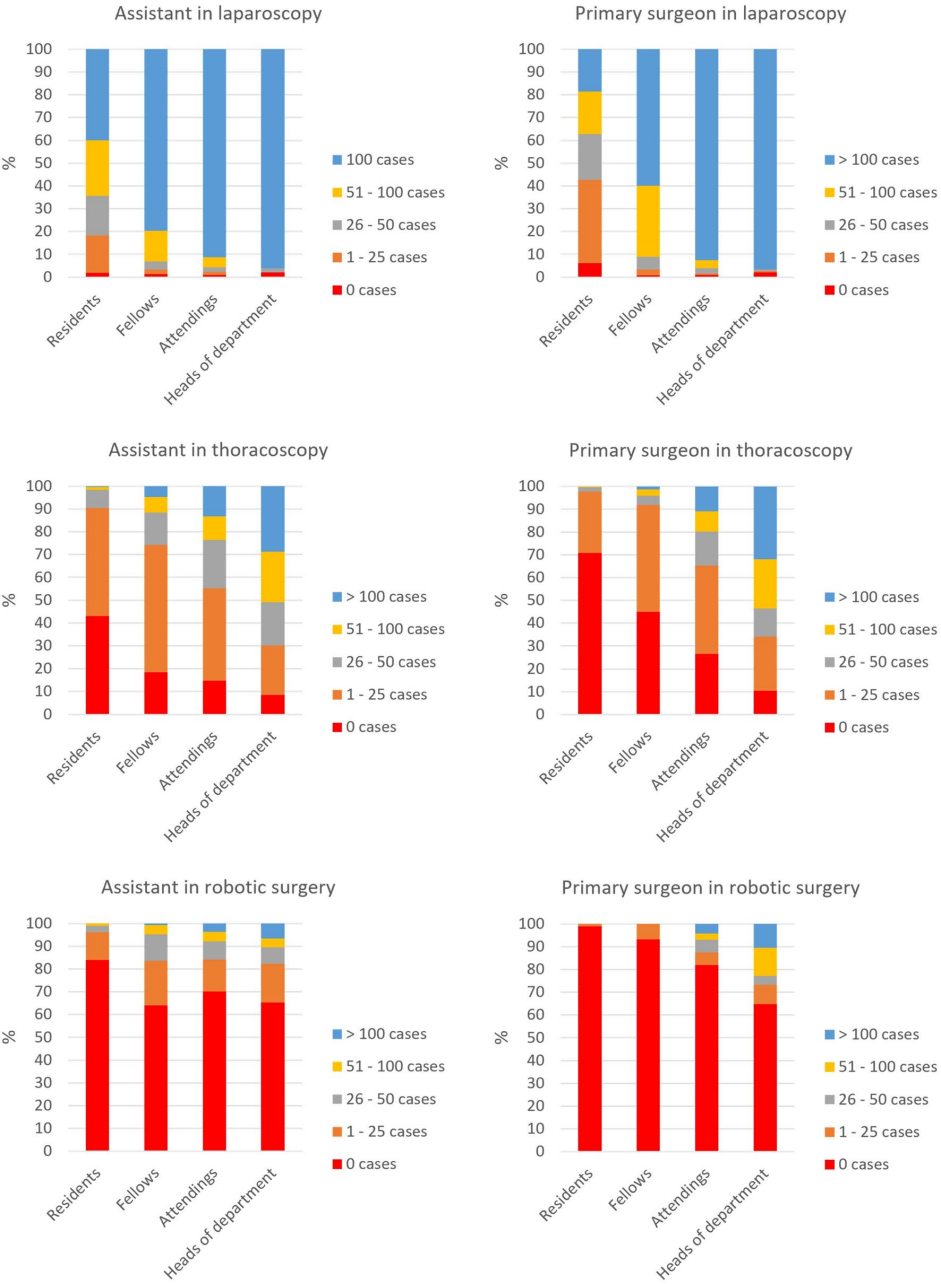
Ratio of operative experience in laparoscopic, thoracoscopic, and robotic cases as assistant or primary surgeon

Detailed characteristics of the cases performed as a primary surgeon or assistant are shown in Fig. 2 and Supplementary Material for each hierarchy level individually.

### MIS training equipment and training time

Over half of the participants (n = 579; 57.4%) reported having access to MIS training equipment at their hospital (Table 2 and Supplementary Material). A box or pelvic trainer (n = 511; 88.3%) was the most frequently used training tool, while the endoscope and camera module (n = 185; 32%) were less frequently used. Virtual reality simulators for laparoscopic or thoracoscopic surgery (n = 132; 22.8%) and robot-assisted surgery (n = 115; 19.9%) were less commonly available training options. Surgical training involving animal or human organs (n = 61; 10.5%) or animals or body donors (n = 24; 4.1%) was rarely offered. Only a small number of participants (n = 78; 7.7%) were allowed to spend time during working hours for MIS training, and these participants spent a median of two hours per week (IQR 1–3) on MIS training.

**Table 2:** Availability and quality of training equipment, training curricula and time for training as reported by participants

	n (% of all)	% (within subcohort)	Median (25–75 Perc.)
*Equipment for MIS-training*	579 (57.4)	100	
Box-/Pelvi-Trainer	511 (50.7)	93.7	
Endoscope	185 (18.4)	79.3	
VR-Trainer	132 (13.1)	80.3	
Robotic VR-Trainer	115 (11.4)	27	
Wet-lab (animal organs)	61 (6.1)	21.7	
Wet-lab (whole animals/body donors)	24 (2.4)	23.3	
Other	22 (2.2)	4.7	
*Skills lab training curriculum*	300 (29.8)	100	
Laparoscopic basic skills	281 (27.9)	88.3	
Laparoscopic surgery sub-steps	238 (23.6)	32	
Laparoscopic assistence	241 (23.9)	22.8	
Robotic basic skills	81 (8)	19.9	
Robotic surgery sub-steps	65 (6.4)	10.5	
Robotic assistence	70 (6.9)	4.1	
Other	14 (1.4)	3.8	
*Intraoperative training curriculum*	262 (26)	100	
Laparoscopic surgery sub-steps	251 (24.9)	95.8	
Laparoscopic assistence	243 (24.1)	92.7	
Robotic surgery sub-steps	49 (4.9)	18.7	
Robotic assistence	54 (5.4)	20.6	
Other	5 (0.5)	1.9	
*Time dedicated for training*	78 (7.7)		
Training hours (hours/week)			2 (1–3)

### Skills lab MIS training

A skills lab training curriculum outside the OR existed in 300 cases (29.8%) (Table 2 and Supplementary Material). The most frequently included skills were basic laparoscopic skills (n = 281; 93.7%), such as instrument handling and simple suturing exercises. Simulated surgical steps or complete surgeries (n = 238; 79.3%) and practicing surgical assistance (n = 241; 80.3%) were also commonly included. A small proportion of participants reported that the curriculum included basic robotic skills (n = 81; 27%), surgical steps for robot-assisted procedures (n = 65; 21.7%), or surgical assistance (n = 70; 23.3%) in robot-assisted surgery.

### Intraoperative MIS training

Among the participants, a quarter (n = 262; 26%) reported that their clinic or department had implemented an intraoperative MIS training curriculum (Table 2 and Supplementary Material). These curricula primarily focused on laparoscopic or thoracoscopic assistance (n = 243; 92.7%) and learning surgical steps (n = 251; 95.8%). Only a very few participants had access to an intraoperative training curriculum for assisting (n = 54; 20.6%) and performing (n = 49; 18.7%) robot-assisted surgery.

### Subcohort analysis for MIS centers and robot-assisted surgery

An additional subcohort analysis including only participants who reported performing robotic procedures in their department or working in a certified MIS center is included in the supplementary material. Satisfaction with MIS training.

On a scale ranging from 0 (not satisfied) to 100 (very satisfied), participants expressed their satisfaction with the MIS training equipment and training times, the quality of skills lab or intraoperative MIS training with a median of 40 (IQR 11–65), 20 (IQR 3–50), 50 (IQR 21–75), and 58 (IQR 30–79), respectively (Supplementary material).

### Factors determining satisfaction with skills lab MIS training

The study also investigated the contributing factors for satisfaction levels with skills lab and intraoperative MIS training for all surgeons, as well as trainees (residents and fellows, n = 410) and teachers (attendings and heads of departments, n = 594) groups individually.

#### All surgeons (Table 3)

A linear logistic regression model was used to analyze the factors that influenced the satisfaction levels of all surgeons. The multivariate analysis showed that several factors had an impact on their satisfaction. Among department-related factors, the number of beds (β − 0.137; 95% confidence intervals [CI] − 0.215 to − 0.059; p < 0.001) was negatively associated with the satisfaction levels of surgeons. Conversely, the number of surgeons performing MIS regularly (β 0.292; 95% CI 0.077 to 0.508; p = 0.008), access to MIS training equipment (β 5.246; 95% CI 1.171 to 9.321; p = 0.012), existence of a skills lab MIS training curriculum (β 12.572; 95% CI 7.263 to 17.881; p < 0.001), an intraoperative MIS training curriculum (β 6.24; 95% CI 0.822 to 11.658; p = 0.024), and time for training (β 7.644; 95% CI 0.915 to 14.374; p = 0.026) were significant positive factors. Performing laparoscopies as the first surgeon (β 3.254; 95% CI 0.472 to 6.036; p = 0.022) and being assistant in robot-assisted surgery (β 2.948; 95% CI 0.327 to 5.57; p = 0.028) also had a positive influence on satisfaction levels. Eventually, the hierarchy status (β 3.379; 95% CI 0.899 to 5.859; p = 0.008) was significantly associated with satisfaction levels.

**Table 3:** Linear regression analysis for all surgeons’ satisfaction with the skill lab MIS training situation (significant p-values marked bold)

	Univariate analysis				Multivariate analysis			
	Coefficient B	S.E	p-value	95% CI	Coefficient B	S.E	p-value	95% CI
Years of practice	0.765	0.095	** < 0.001**	0.579 to 0.951	− 0.046	0.171	0.789	− 0.382 to 0.291
Hierarchy group	6.364	0.644	** < 0.001**	5.101 to 7.627	3.379	1.263	**0.008**	0.899 to 5.859
Number of beds	− 0.082	0.036	**0.023**	− 0.153 to − 0.011	− 0.137	0.04	** < .001**	− 0.215 to -0.059
Number of surgeons employed	− 0.059	0.036	0.107	− 0.13 to 0.013				
All elective surgery	0.246	0.104	**0.018**	0.042 to 0.45	− 0.037	0.111	0.742	− 0.254 to 0.181
Robotic surgery	0.111	0.5	0.824	− 0.869 to 1.092				
Oncological resections	0.173	0.191	0.364	− 0.201 to 0.548				
Emergency surgery	0.234	0.214	0.275	− 0.186 to 0.654				
Number of surgeons performing MIS regularly (min. 1/week)	0.293	0.11	**0.008**	0.078 to 0.508	0.292	0.11	**0.008**	0.077 to 0.508
Certified center for MIS	− 3.064	1.195	**0.01**	− 5.408 to − 0.72	0.064	1.387	0.963	− 2.659 to 2.786
Hands-on training curriculum	23.314	1.954	** < 0.001**	19.479 to 27.149	12.572	2.705	** < 0.001**	7.263 to 17.881
Intraoperative training curriculum	21.612	2.084	** < 0.001**	17.523 to 25.701	6.24	2.76	**0.024**	0.822 to 11.658
Access to training equipment	12.927	1.921	** < 0.001**	9.158 to 16.696	5.246	2.076	**0.012**	1.171 to 9.321
Dedicated time for training during working hours	23.399	3.478	** < 0.001**	16.573 to 30.224	7.644	3.428	**0.026**	0.915 to 14.374
Experience laparoscopic assistant	6.144	1.013	** < 0.001**	4.157 to 8.131	− 1.212	1.559	0.437	− 4.272 to 1.847
Experience laparoscopic first surgeon	6.46	0.814	** < 0.001**	4.861 to 8.058	3.254	1.417	**0.022**	0.472 to 6.036
Experience thoracoscopic assistant	4.296	0.755	** < 0.001**	2.814 to 5.777	0.971	1.402	0.489	− 1.782 to 3.724
Experience thoracoscopic first surgeon	4.87	0.721	** < 0.001**	3.455 to 6.286	− 0.405	1.489	0.786	− 3.329 to 2.518
Experience robotic assistant	5.44	0.978	** < 0.001**	3.52 to 7.36	2.948	1.336	**0.028**	0.327 to 5.57
Experience robotic first surgeon	6.14	0.99	** < 0 .001**	4.198 to 8.082	0.081	1.329	0.951	− 2.528 to 2.691

#### Trainee group (n = 410) (Table 4)

Analyzing the hierarchy groups separately, the multivariate analysis for significant factors influencing the satisfaction level of trainees with skills lab MIS training revealed that a higher number of surgeons employed at the department (β, − 0.452; 95% CI − 0.796 to − 0.108; p = 0.01) resulted in lower satisfaction levels within the trainee group. However, the existence of a skills lab MIS training curriculum (β 21.017; 95% CI 12.231 to 29.803; p < 0.001), an intraoperative MIS training curriculum (β 14.807; 95% CI 4.84 to 24.774; p = 0.004), and access to MIS training equipment (β 8.1; 95% CI 1.808 to 14.391; p = 0.012) were strong beneficial factors. Moreover, experience as the first surgeon in laparoscopic surgeries (β 3.741; 95% CI 0.797 to 6.686; p = 0.013) and as an assistant in robot-assisted surgeries (β 6.449; 95% CI 2.191 to 10.707; p = 0.003) also had a significantly positive influence on the satisfaction levels.

**Table 4:** Linear regression analysis for trainee’ satisfaction with the skills lab MIS training situation (significant p-values marked bold)

	Univariate analysis				Multivariate analysis			
	Coefficient B	S.E	p-value	95% CI	Coefficient B	S.E	p-value	95% CI
Years of practice	− 0.557	0.351	0.114	− 1.248 to 0.134				
Hierarchy group	2.383	3.148	0.45	− 3.806 to 8.572				
Number of beds	− 0.13	0.054	**0.016**	− 0.236 to − 0.024	− 0.028	0.086	0.744	− 0.198 to 0.141
Number of surgeons employed	− 0.336	0.098	** < .001**	− 0.529 to − 0.143	− 0.452	0.175	**0.01**	− 0.796 to − 0.108
All elective surgery	0.236	0.158	0.135	− 0.074 to 0.546				
Robotic surgery	− 1.284	0.72	0.075	− 2.7 to 0.131				
Oncological resections	− 0.151	0.365	0.679	− 0.869 to 0.566				
Emergency surgery	0.683	0.414	0.1	− 0.131 to 1.497				
Number of surgeons performing MIS regularly (min. 1/week)	0.517	0.266	0.052	− 0.006 to 1.039				
Certified center for MIS	− 0.511	1.775	0.774	− 4 to 2.979				
Hands-on training curriculum	29.186	3.668	** < 0.001**	21.973 to 36.4	21.017	4.465	** < 0.001**	12.231 to 29.803
Intraoperative training curriculum	29.852	4.131	** < 0.001**	21.728 to 37.977	14.807	5.065	**0.004**	4.84 to 24.774
Access to training equipment	10.627	3.058	** < 0.001**	4.615 to 16.64	8.1	3.198	**0.012**	1.808 to 14.391
Dedicated time for training during working hours	41.86	7.79	** < 0.001**	26.542 to 57.177	10.467	7.801	0.181	− 4.884 to 25.818
Experience laparoscopic assistant	3.953	1.338	**0.003**	1.323 to 6.584	− 1.42	1.741	0.416	− 4.846 to 2.006
Experience laparoscopic first surgeon	4.026	1.159	** < 0.001**	1.747 to 6.305	3.741	1.496	**0.013**	0.797 to 6.686
Experience thoracoscopic assistant	1.063	1.764	0.547	− 2.405 to 4.532				
Experience thoracoscopic first surgeon	2.635	2.293	0.251	− 1.872 to 7.141				
Experience robotic assistant	6.086	2.109	**0.004**	1.94 to 10.232	6.449	2.164	**0.003**	2.191 to 10.707
Experience robotic first surgeon	10.123	8.609	0.24	− 6.8 to 27.046				

#### Teacher group (n = 594) (Table 5)

The multivariate analysis showed that the number of employed surgeons (β 0.233; 95% CI 0.019 to 0.446; p = 0.033), the existence of a skills lab MIS training curriculum (β 9.258; 95% CI 2.736 to 15.78; p = 0.005), and the hierarchical status (β 3.891; 95% CI 0.387 to 7.395; p = 0.03) remained significant factors influencing the satisfaction levels of the teachers with skills lab MIS training.

**Table 5:** Linear regression analysis for teachers’ satisfaction with the skills lab MIS training situation (significant p-values marked bold)

	Univariate analysis				Multivariate analysis			
	Coefficient B	S.E	p-value	95% CI	Coefficient B	S.E	p-value	95% CI
Years of practice	0.518	0.144	** < 0.001**	0.236 to 0.8	0.189	0.179	0.293	− 0.164 to 0.541
Hierarchy group	6.715	1.375	** < 0.001**	4.014 to 9.416	3.891	1.784	**0.03**	0.387 to 7.395
Number of beds	0.036	0.046	0.439	− 0.055 to 0.127				
Number of surgeons employed	0.008	0.036	0.822	− 0.063 to 0.079				
All elective surgery	0.242	0.13	0.062	− 0.012 to 0.497				
Robotic surgery	1.832	0.646	**0.005**	0.563 to 3.101	− 0.266	0.872	0.76	− 1.978 to 1.446
Oncological resections	0.371	0.208	0.075	− 0.038 to 0.78				
Emergency surgery	0.049	0.236	0.834	− 0.414 to 0.512				
Number of surgeons performing MIS regularly (min. 1/week)	0.253	0.111	**0.023**	0.034 to 0.472	0.233	0.109	**0.033**	0.019 to 0.446
Certified MIS center	3.061	1.794	0.089	− 0.463 to 6.585				
Hands-on training curriculum	16.606	2.271	** < 0.001**	12.145 to 21.067	9.258	3.32	**0.005**	2.736 to 15.78
Intraoperative training curriculum	14.217	2.38	** < 0.001**	9.543 to 18.891	3.321	3.23	0.304	− 3.024 to 9.666
Access to training equipment	12.028	2.338	** < 0.001**	7.437 to 16.619	4.16	2.63	0.114	− 1.007 to 9.327
Dedicated time for training during working hours	14.082	3.654	** < 0.001**	6.905 to 21.258	4.884	3.764	0.195	− 2.511 to 12.278
Experience laparoscopic assistant	1.687	1.878	0.369	− 2.002 to 5.375				
Experience laparoscopic first surgeon	1.52	1.98	0.443	− 2.368 to 5.409				
Experience thoracoscopic assistant	2.322	0.876	**0.008**	0.602 to 4.043	2.388	1.667	0.153	− 0.887 to 5.664
Experience thoracoscopic first surgeon	2.452	0.824	**0.003**	0.834 to 4.07	− 1.769	1.664	0.288	− 5.038 to 1.501
Experience robotic assistant	3.858	1.044	** < 0.001**	1.808 to 5.908	1.904	1.66	0.252	− 1.357 to 5.165
Experience robotic first surgeon	3.949	0.964	** < 0.001**	2.057 to 5.842	0.66	1.499	0.66	− 2.284 to 3.604

### Factors determining satisfaction with intraoperative MIS training

#### All surgeons (Table 6)

In the multivariate analysis for factors influencing the satisfaction with intraoperative MIS training among all surgeons, significant factors included access to MIS training equipment (β 4.295; 95% CI 0.252 to 8.339; p = 0.037), offering an intraoperative MIS training curriculum (β 16.541; 95% CI 11.183 to 21.899; p < 0.001), and experience as the first surgeon in laparoscopic surgery (β 3.748; 95% CI 1.007 to 6.489; p = 0.007).

**Table 6:** Linear regression analysis for all surgeons’ satisfaction with the intraoperative training situation (significant p-values marked bold)

	Univariate analysis				Multivariate analysis			
	Coefficient B	S.E	p-value	95% CI	Coefficient B	S.E	p-value	95% CI
Years of practice	0.725	0.092	** < 0.001**	0.545 to 0.905	0.007	0.169	0.969	− 0.325 to 0.338
Hierarchy group	5.521	0.627	** < 0.001**	4.291 to 6.752	2.426	1.243	0.051	− 0.015 to 4.866
Number of beds	− 0.132	0.035	** < 0.001**	− 0.2 to − 0.064	− 0.045	0.054	0.409	− 0.15 to 0.061
Number of surgeons employed	− 0.094	0.035	**0.007**	− 0.162 to − 0.025	− 0.187	0.105	0.074	− 0.393 to 0.018
All elective surgery	0.234	0.1	**0.02**	0.038 to 0.431	0.137	0.106	0.198	− 0.072 to 0.346
Robotic surgery	− 1.032	0.481	**0.032**	− 1.977 to − 0.088	− 1.119	0.622	0.072	− 2.34 to 0.102
Oncological resections	0.071	0.184	0.698	− 0.29 to 0.433				
Emergency surgery	0.138	0.207	0.503	− 0.267 to 0.544				
Number of surgeons performing MIS regularly (min. 1/week)	0.202	0.106	0.056	− 0.006 to 0.41				
Certified MIS center	− 2.363	1.155	**0.041**	− 4.629 to − 0.097	0.912	1.369	0.506	− 1.776 to 3.599
Hands-on training curriculum	17.219	1.948	** < 0.001**	13.397 to 21.042	1.352	2.67	0.613	− 3.889 to 6.592
Intraoperative training curriculum	23.586	1.981	** < 0.001**	19.699 to 27.474	16.541	2.73	** < 0.001**	11.183 to 21.899
Access to training equipment	9.115	1.874	** < 0.001**	5.437 to 12.793	4.295	2.06	**0.037**	0.252 to 8.339
Dedicated time for training during working hours	21.115	3.382	** < 0.001**	14.479 to 27.752	6.302	3.391	0.063	− 0.353 to 12.957
Experience laparoscopic assistant	5.654	0.979	** < 0.001**	3.732 to 7.575	− 1.549	1.543	0.316	− 4.578 to 1.479
Experience laparoscopic first surgeon	5.804	0.79	** < 0.001**	4.254 to 7.353	3.748	1.396	**0.007**	1.007 to 6.489
Experience thoracoscopic assistant	3.089	0.734	** < 0.001**	1.648 to 4.53	0.062	1.383	0.964	− 2.654 to 2.777
Experience thoracoscopic first surgeon	3.897	0.701	** < 0.001**	2.522 to 5.273	− 0.028	1.47	0.985	− 2.913 to 2.856
Experience robotic assistant	1.964	0.957	**0.04**	0.086 to 3.842	1.931	1.401	0.169	− 0.82 to 4.681
Experience robotic first surgeon	3.358	0.968	** < 0.001**	1.459 to 5.258	0.008	1.32	0.995	− 2.583 to 2.599

#### Trainee group (n = 410) (Table 7)

The number of surgeons employed at the department (β − 0.57; 95% CI − 0.929 to − 0.21; p = 0.002) was a negative factor on satisfaction in the trainee group. On the other hand, the number of surgeons performing MIS regularly (β 1.087; 95% CI 0.407 to 1.767; p = 0.002), the existence of a skills lab MIS training curriculum (β 10.1; 95% CI 1.031 to 19.17; p = 0.029), an existing intraoperative MIS training curriculum (β 23.061; 95% CI 12.768 to 33.353; p < 0.001), and MIS training equipment (β 7.3; 95% CI 0.834 to 13.766; p = 0.027) significantly improved the satisfaction of the trainees with intraoperative MIS training.

**Table 7:** Linear regression analysis for trainees’ satisfaction with the intraoperative training situation (significant p-values marked bold)

	Univariate analysis				Multivariate analysis			
	Coefficient B	S.E	p-value	95% CI	Coefficient B	S.E	p-value	95% CI
Years of practice	− 0.346	0.352	0.325	− 1.038 to 0.345				
Hierarchy group	1.984	3.146	0.529	− 4.2 to 8.168				
Number of beds	− 0.17	0.053	0.002	− 0.275 to − 0.065	− 0.034	0.091	0.712	− 0.213 to 0.146
Number of surgeons employed	− 0.464	0.097	< 0.001	− 0.654 to − 0.275	− 0.57	0.183	**0.002**	− 0.929 to − 0.21
All elective surgery	0.321	0.157	0.041	0.012 to 0.63	0.136	0.183	0.457	− 0.224 to 0.496
Robotic surgery	− 1.829	0.716	0.011	− 3.237 to − 0.421	− 0.747	0.814	0.36	− 2.349 to 0.855
Oncological resections	− 0.151	0.365	0.679	− 0.868 to 0.566				
Emergency surgery	0.838	0.413	0.043	0.026 to 1.65	− 0.022	0.475	0.963	− 0.957 to 0.913
Number of surgeons performing MIS regularly (min. 1/week)	0.698	0.264	0.009	0.178 to 1.218	1.087	0.345	**0.002**	0.407 to 1.767
Certified MIS center	− 0.38	1.773	0.83	− 3.866 to 3.106				
Hands-on training curriculum	21.37	3.823	< 0.001	13.852 to 28.887	10.1	4.609	**0.029**	1.031 to 19.17
Intraoperative training curriculum	32.981	4.075	< .001	24.966 to 40.996	23.061	5.231	** < 0.001**	12.768 to 33.353
Access to training equipment	8.35	3.08	0.007	2.295 to 14.405	7.3	3.286	**0.027**	0.834 to 13.766
Dedicated time for training during working hours	40.761	7.853	< 0.001	25.32 to 56.202	12.752	8.01	0.112	− 3.01 to 28.515
Experience laparoscopic assistant	3.609	1.339	0.007	0.976 to 6.241	− 2.101	1.8	0.244	− 5.642 to 1.44
Experience laparoscopic first surgeon	3.481	1.163	0.003	1.196 to 5.766	3.037	1.547	0.051	− 0.007 to 6.081
Experience thoracoscopic assistant	0.961	1.763	0.586	− 2.504 to 4.427				
Experience thoracoscopic first surgeon	2.927	2.289	0.202	− 1.573 to 7.427				
Experience robotic assistant	3.331	2.122	0.117	− 0.84 to 7.503				
Experience robotic first surgeon	6.293	8.608	0.465	− 10.629 to 23.216				

#### Teacher group (n = 594) (Table 8)

The only significant factor improving satisfaction within the teacher group was the existence of an intraoperative training curriculum (β 13.803; 95% CI 7.708 to 19.898; p < 0.001).

**Table 8:** Linear regression analysis for teachers’ satisfaction with the intraoperative training situation (significant p-values marked bold)

	Univariate analysis				Multivariate analysis			
	Coefficient B	S.E	p-value	95% CI	Coefficient B	S.E	p-value	95% CI
Years of practice	0.54	0.136	** < 0.001**	0.273 to 0.807	0.268	0.162	0.099	− 0.05 to 0.587
Hierarchy group	5.093	1.313	** < 0.001**	2.515 to 7.672	2.243	1.569	0.153	− 0.839 to 5.325
Number of beds	− 0.028	0.044	0.527	− 0.114 to 0.058				
Number of surgeons employed	− 0.013	0.034	0.7	− 0.081 to 0.054				
All elective surgery	0.141	0.123	0.252	− 0.101 to 0.383				
Robotic surgery	0.086	0.617	0.889	− 1.125 to 1.297				
Oncological resections	0.236	0.198	0.232	− 0.152 to 0.624				
Emergency surgery	− 0.105	0.223	0.64	− 0.543 to 0.334				
Number of surgeons performing MIS regularly (min. 1/week)	0.108	0.106	0.31	− 0.1 to 0.316				
Certified MIS center	3.349	1.699	**0.049**	0.012 to 6.687	3.492	1.962	0.076	− 0.362 to 7.347
Hands-on training curriculum	11.016	2.205	** < 0.001**	6.685 to 15.348	− 1.862	3.171	0.557	− 8.092 to 4.368
Intraoperative training curriculum	16.086	2.224	** < 0.001**	11.718 to 20.454	13.803	3.103	** < 0.001**	7.708 to 19.898
Access to training equipment	7.7	2.238	** < 0.001**	3.304 to 12.096	1.901	2.467	0.441	− 2.944 to 6.747
Dedicated time for training during working hours	12.166	3.485	** < 0.001**	5.321 to 19.01	4.255	3.596	0.237	− 2.808 to 11.318
Experience laparoscopic assistant	2.039	1.779	0.252	− 1.456 to 5.533				
Experience laparoscopic first surgeon	2.024	1.876	0.281	− 1.66 to 5.707				
Experience thoracoscopic assistant	0.867	0.834	0.299	− 0.771 to 2.506				
Experience thoracoscopic first surgeon	1.373	0.784	0.08	− 0.167 to 2.914				
Experience robotic assistant	0.32	1.001	0.749	− 1.645 to 2.286				
Experience robotic first surgeon	1.297	0.925	0.161	− 0.519 to 3.112				

## Discussion

MIS is no longer considered an exotic or specialized surgical approach. At academic centers, MIS is being used for 94% of bariatric procedures, 83.7% of anti-reflux surgery, 77.1% of cholecystectomies, and 79.2% of appendectomies [8]. In Germany, appendectomies were performed laparoscopically in as many as 85% of cases [17]. However, the utilization of MIS varies widely, leading to a potential inequality in surgical care [18]. The ultimate goal should be to offer MIS to most patients and have most surgeons perform it. This requires thorough MIS education and training for all surgeons, especially since MIS requires an extended skill set including hand-eye-coordination, depth perception, diminished haptic feedback, specialized technological knowledge and surgical planning compared to open surgery.

However, the present survey indicates a different reality. Even though all participants reported using MIS in their department and almost all departments also offered MIS for emergency and oncological procedures, slightly over half (57.4%) of the participants had access to appropriate MIS training equipment, and only 29.8% used a skills lab MIS training curriculum, with a small proportion (7.7%) provided with time to train. The latter may already be a sign that we are already in a vicious cycle in which the shortage of surgical professionals further exacerbates the minimal margin of available time and human resources for training in addition to patient care.

The discrepancy between the availability of MIS training equipment and a corresponding standardized training curriculum has been previously demonstrated by Huber et al. in a survey. The survey reported that 52.8% (n = 140) of participating hospitals had laparoscopic training simulators, but only 43.3% (n = 103) provided a respective curriculum [19].

This concerning lack of training does not seem to be a solely German issue, as Ranjit et al. found that only 8% of first-year residents in the United Kingdom received laparoscopic skills training [20].

Even with existing training equipment, the quality of MIS training in the Skills Lab appears to be limited according to our study, as most reported training devices were box trainers, which are generally not suitable for more advanced training scenarios. Only a few respondents reported access to virtual reality simulators and surgical training opportunities on live animals or cadavers. Both simulation-based and in vivo training are essential for skill transfer to real-world operations [13, 21, 22]. The understanding that training to achieve laparoscopic competence should include realistic training, such as on live animals and cadavers, appears to be present in both residents and instructors [11]. Consequently, as early as 2006, most residents agreed in a survey that simulation training should be mandatory in surgical residency [23].

Despite this, our findings indicate that MIS training is still mainly conducted “on the job” during operations. Controversially, only a quarter of participants reported the existence of a structured intraoperative MIS training curriculum. Such inadequate training can lead to concerning results, as shown by Mattar et al. with 30% of fellows unable to perform a laparoscopic cholecystectomy independently and 56% unable to suture laparoscopically [24].

The question remains as to how this could have happened. Part of the problem is that German board certification for general or abdominal surgery has not yet required participation in an MIS course, nor is participation in laparoscopic or robotic procedures explicitly required. This makes it more difficult for surgeons in training to claim these types of procedures, as their supervisors and “teachers” are not obliged to provide this type of training or procedure.

The data from the present survey suggests that residents and to some extent even fellows are not able to gain sufficient operative experience as operating surgeons in MIS cases. Considering the increased popularity of robot-assisted surgery, the number of surgeons who have already participated in robot-assisted surgery exceeded the number of survey participants reporting robotic training equipment and curriculum by far. A very similar finding, with 60% of residents participating in robot-assisted surgery without having received any formal training, has been demonstrated before by Farivar et al. [25] Nevertheless, the number of operations performed has always been a benchmark in the training of surgeons. Fellow exposure to certain MIS procedures during training has been shown to increase the acceptance of such procedures, leading to a higher likelihood of performing them in the future [26]. Besides, being the operating surgeon and having operative autonomy have been identified as factors related to increased satisfaction among residents [27, 28].

Regarding satisfaction with the MIS training situation, our findings indicate that there are multiple factors influencing the satisfaction of surgeons. However, department-related factors, such as the number of beds and surgeons performing MIS regularly, showed a comparatively weak impact. Exposure to surgery and the existence of training curricula and equipment had the strongest effect on the satisfaction of surgeons with both, skills lab and intraoperative MIS training. Interestingly, satisfaction with skills lab MIS training also depended on hierarchy status.

The issue at hand is that there are disparities between trainees and teachers in their perceptions of different training methods and the quality of education [29–31]. These disparities may lead to different expectations and satisfaction with the training offered depending on the affiliation of the trainee or teacher groups. Our multivariate linear regression analysis for the trainee and teacher groups individually revealed that factors with a significant impact on satisfaction with extra- and intraoperative training were different between the two groups. In general, exposure to surgery played an important and beneficial role in influencing the satisfaction level within the trainee group. Participation in operations was one of the strongest influencing factors, supporting the findings of Ko and Perone. However, both trainees and teachers found the existence of a skills lab or an intraoperative MIS training curriculum, along with access to training equipment, essential for their satisfaction levels. Other significant factors, like department size or the number of surgeons performing MIS regularly, were relevant only for the trainee group and had comparatively weak influences on their satisfaction levels. Interestingly, larger departments with more patient beds and employed surgeons were at a disadvantage. Possible reasons for this include increased competition among employees for participation in operations and a greater number of complex cases beyond the capability of residents. However, the effect of this observation was comparatively weak compared to other factors, such as the existence of a training curriculum.

### Limitations

The present study has several limitations that should be noted. First, the data was obtained through a survey, which can allow for a subjective interpretation of questions and responses. Consequently, the given answers may be biased by the subjective perspectives of respondents. Especially the estimation of surgery numbers per department, employees, and operative experience can be subject to very subjective alterations. However, the larger volume of participants and the even distribution of hierarchy groups provide a good balance for statistical outliers. Still, the generalizability could be compromised. We found 241 participants (23.9%) reporting to work at a certified MIS center but with currently 69 hospitals in Germany being certified as MIS center, we can assume an overrepresentation of MIS centers in our data. This overrepresentation of MIS centers, which must maintain MIS training equipment to obtain certification, may mask an even more worrisome general lack of MIS training availability.

Also, surveys are prone to inviting only a selected group of people of interest, which can lead to self-selection sampling bias. To reduce this bias, the survey was not spread using a surgical association or society by proxy, but all surgical departments and mostly even surgeons individually were contacted using the publicly available contact information. However, this approach meant that a response rate could not be reliably calculated. Finally, common-method bias cannot be completely ruled out, implying that there is a possibility that participants interpreted the underlying motivation of the survey and adjusted their responses accordingly [32].

## Conclusion

This study provides the most comprehensive insight to date into the MIS training landscape in Germany. The results confirm that the predominant training methodology seems to be “training-on-the-job,” with a lack of training equipment, curricula, and dedicated time for training. Further analyses revealed factors with a significant impact on the satisfaction of surgeons with the respective training situations.

The lack of standardized MIS training in Germany, both in skills labs and in the OR, is worrysome. A potential cause might be the missing obligation to participate in MIS training and maybe even more the lack of obligation to teach sufficiently and to provide training time. As a consequence, it should be considered to include a mandatory participation in MIS training and MIS procedures to the requirements for the German surgical board certification. Furthermore, targeted interventions could be developed based on the results to improve surgeon well-being, increase surgical residency application rates, or avoid dropouts.

### Supplementary Information

Below is the link to the electronic supplementary material.

Supplementary file1 (JPG 211 KB

Supplementary file2 (DOCX 25 KB)

Supplementary file3 (DOCX 22 KB)

Supplementary file4 (DOCX 23 KB)

Supplementary file5 (DOCX 19 KB)
