# Supplementary figures and images for: Satisfaction of surgeons with the current state of training in minimally invasive surgery: a survey among German surgeons
Source: Surg Endosc. 2023 Dec 12;38(2):1029–44. doi: 10.1007/s00464-023-10584-y (PMC10830590; doi:10.1007/s00464-023-10584-y)

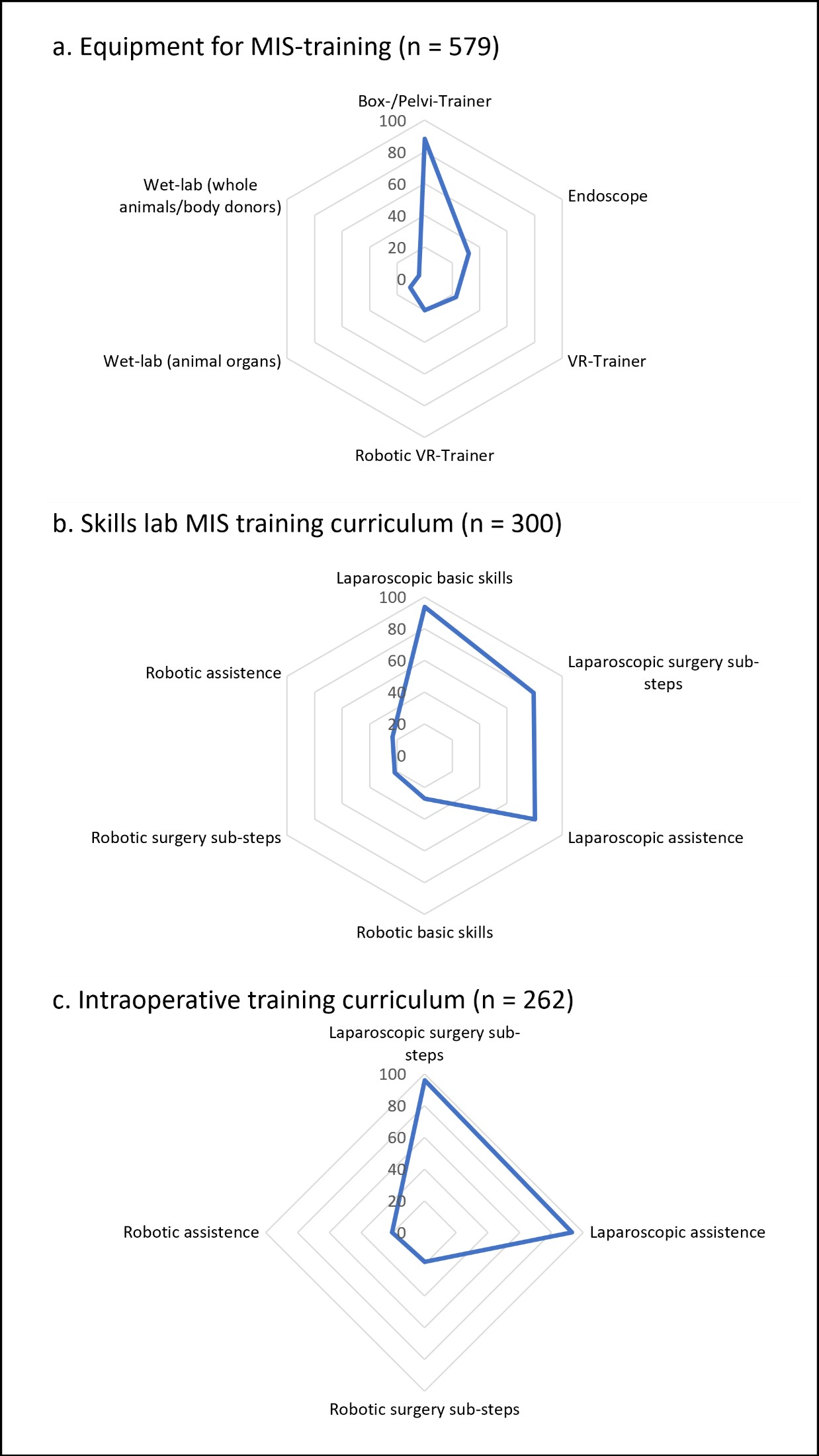

Supplement: Supplementary file 1 — Supplementary file1 (JPG 211 KB [file 464_2023_10584_MOESM1_ESM.jpg]
