# Supplementary material for: Satisfaction of surgeons with the current state of training in minimally invasive surgery: a survey among German surgeons
Source: Surg Endosc. 2023 Dec 12;38(2):1029–44. doi: 10.1007/s00464-023-10584-y (PMC10830590; doi:10.1007/s00464-023-10584-y)
Supplement: Supplementary file 2 — Supplementary file2 (DOCX 25 KB) [file 464_2023_10584_MOESM2_ESM.docx]

|  | **Laparoscopic surgery** | | | | | | | | | |
| --- | --- | --- | --- | --- | --- | --- | --- | --- | --- | --- |
|  | **assistant** | | | | | **primary surgeon** | | | | |
|  | **0 cases** | **1 - 25 cases** | **26 - 50 cases** | **51 - 100 cases** | **> 100 cases** | **0 cases** | **1 - 25 cases** | **26 - 50 cases** | **51 - 100 cases** | **> 100 cases** |
|  | **n (%)** | **n (%)** | **n (%)** | **n (%)** | **n (%)** | **n (%)** | **n (%)** | **n (%)** | **n (%)** | **n (%)** |
| **Residents** | 5 (1.9) | 43 (16.3) | 46 (17.5) | 64 (24.3) | 105 (39.9) | 16 (6.1) | 96 (36.5) | 53 (20.2) | 49 (18.6) | 49 (18.6) |
| **Fellows** | 2 (1.4) | 3 (2) | 5 (3.4) | 20 (13.6) | 117 (79.6) | 1 (0.7) | 4 (2.7) | 8 (5.4) | 46 (31.3) | 88 (59.9) |
| **Attendings** | 4 (0.9) | 6 (1.4) | 9 (2) | 19 (4.3) | 403 (91.4) | 4 (0.9) | 3 (0.7) | 10 (2.3) | 16 (3.6) | 408 (92.5) |
| **Heads of department** | 3 (2) | 0 (0) | 3 (2) | 0 (0) | 147 (96.1) | 3 (2) | 1 (0.7) | 1 (0.7) | 0 (0) | 148 (96.7) |
| **Others** | 0 (0) | 0 (0) | 0 (0) | 0 (0) | 4 (100) | 0 (0) | 1 (25) | 0 (0) | 0 (0) | 3 (75) |
|  |  |  |  |  |  |  |  |  |  |  |
|  | **Thoracoscopic surgery** | | | | | | | | | |
|  | **assistant** | | | | | **primary surgeon** | | | | |
|  | **0 cases** | **1 - 25 cases** | **26 - 50 cases** | **51 - 100 cases** | **> 100 cases** | **0 cases** | **1 - 25 cases** | **26 - 50 cases** | **51 - 100 cases** | **> 100 cases** |
|  | **n (%)** | **n (%)** | **n (%)** | **n (%)** | **n (%)** | **n (%)** | **n (%)** | **n (%)** | **n (%)** | **n (%)** |
| **Residents** | 113 (43) | 125 (47.5) | 21 (8) | 3 (1.1) | 1 (0.4) | 186 (70.7) | 71 (27) | 5 (1.9) | 1 (0.4) | 0 (0) |
| **Fellows** | 27 (18.4) | 82 (55.8) | 21 (14.3) | 10 (6.8) | 7 (4.8) | 66 (44.9) | 69 (46.9) | 6 (4.1) | 4 (2.7) | 2 (1.4) |
| **Attendings** | 65 (14.7) | 178 (40.4) | 94 (21.3) | 46 (10.4) | 58 (13.2) | 117 (26.5) | 171 (38.8) | 66 (15) | 39 (8.8) | 48 (10.9) |
| **Heads of department** | 13 (8.5) | 33 (21.6) | 29 (19) | 34 (22.2) | 44 (28.8) | 16 (10.5) | 36 (23.5) | 19 (12.4) | 33 (21.6) | 49 (32) |
| **Others** | 0 (0) | 1 (25) | 0 (0) | 2 (50) | 1 (25) | 1 (25) | 0 (0) | 0 (0) | 2 (50) | 1 (25) |
|  |  |  |  |  |  |  |  |  |  |  |
|  | **Robotic surgery** | | | | | | | | | |
|  | **assistant** | | | | | **primary surgeon** | | | | |
|  | **0 cases** | **1 - 25 cases** | **26 - 50 cases** | **51 - 100 cases** | **> 100 cases** | **0 cases** | **1 - 25 cases** | **26 - 50 cases** | **51 - 100 cases** | **> 100 cases** |
|  | **n (%)** | **n (%)** | **n (%)** | **n (%)** | **n (%)** | **n (%)** | **n (%)** | **n (%)** | **n (%)** | **n (%)** |
| **Residents** | 221 (84) | 32 (12.2) | 7 (2.7) | 3 (1.1) | 0 (0) | 260 (98.9) | 3 (1.1) | 0 (0) | 0 (0) | 0 (0) |
| **Fellows** | 94 (63.9) | 29 (19.7) | 17 (11.6) | 6 (4.1) | 1 (0.7) | 137 (93.2) | 10 (6.8) | 0 (0) | 0 (0) | 0 (0) |
| **Attendings** | 309 (70.1) | 62 (14.1) | 35 (7.9) | 19 (4.3) | 16 (3.6) | 361 (81.9) | 25 (5.7) | 24 (5.4) | 12 (2.7) | 19 (4.3) |
| **Heads of department** | 100 (65.4) | 26 (17) | 11 (7.2) | 6 (3.9) | 10 (6.5) | 99 (64.7) | 13 (8.5) | 6 (3.9) | 19 (12.4) | 16 (10.5) |
| **Others** | 3 (75) | 1 (25) | 0 (0) | 0 (0) | 0 (0) | 4 (100) | 0 (0) | 0 (0) | 0 (0) | 0 (0) |

Supplementary Material Table 1: Operative experience for laparoscopic, thoracoscopic and robotic surgery for each hierarchy group
