# Supplementary material for: Satisfaction of surgeons with the current state of training in minimally invasive surgery: a survey among German surgeons
Source: Surg Endosc. 2023 Dec 12;38(2):1029–44. doi: 10.1007/s00464-023-10584-y (PMC10830590; doi:10.1007/s00464-023-10584-y)
Supplement: Supplementary file 3 — Supplementary file3 (DOCX 22 KB) [file 464_2023_10584_MOESM3_ESM.docx]

| **Subcohort of participants performing robot-assisted surgery at their department (n = 353)** | | **n (% of all)** | **% (within subcohort)** | **Median (25. - 75. Perc.)** |
| --- | --- | --- | --- | --- |
| **Equipment for MIS-training** | | 259 (73.1) | 100 |  |
|  | Robotic VR-Trainer | 114 (32.3) | 44 |  |
|  | Wet-lab (animal organs) | 37 (10.5) | 14.3 |  |
|  | Wet-lab (whole animals/body donors) | 14 (4) | 5.4 |  |
| **Skills lab training curriculum** | | 124 (35.1) | 100 |  |
|  | Robotic basic skills | 76 (21.5) | 61.3 |  |
|  | Robotic surgery sub-steps | 59 (16.7) | 47.6 |  |
|  | Robotic assistence | 65 (18.4) | 52.4 |  |
| **Intraoperative training curriculum** | | 97 (27.5) | 100 |  |
|  | Robotic surgery sub-steps | 47 (13.3) | 48.4 |  |
|  | Robotic assistence | 52 (14.7) | 53.6 |  |
| **Time dedicated for training** | | 28 (7.9) |  |  |
|  | Training hours [hours/week] |  |  | 2 (2-4) |

Supplementary Material Table 2: Subcohort analysis considering only participants reporting robot-assisted surgery at their department
