# Supplementary material for: Satisfaction of surgeons with the current state of training in minimally invasive surgery: a survey among German surgeons
Source: Surg Endosc. 2023 Dec 12;38(2):1029–44. doi: 10.1007/s00464-023-10584-y (PMC10830590; doi:10.1007/s00464-023-10584-y)
Supplement: Supplementary file 4 — Supplementary file4 (DOCX 23 KB) [file 464_2023_10584_MOESM4_ESM.docx]

| **Subcohort of participants working at a certified MIS center (n = 241)** | | **n (% of all)** | **% (within subcohort)** | **Median (25. - 75. Perc.)** |
| --- | --- | --- | --- | --- |
| **Equipment for MIS-training** | | 175 (72.6) | 100 |  |
|  | Box-/Pelvi-Trainer | 158 (65.6) | 90.3 |  |
|  | Endoscope | 59 (24.5) | 33.7 |  |
|  | VR-Trainer | 40 (16.6) | 22.9 |  |
|  | Robotic VR-Trainer | 40 (16.6) | 22.9 |  |
|  | Wet-lab (animal organs) | 30 (12.4) | 17.1 |  |
|  | Wet-lab (whole animals/body donors) | 12 (5) | 6.9 |  |
|  | Other | 9 (3.7) | 5.1 |  |
| **Skills lab training curriculum** | | 102 (42.3) | 100 |  |
|  | Laparoscopic basic skills | 98 (40.7) | 96.1 |  |
|  | Laparoscopic surgery sub-steps | 82 (34) | 80.4 |  |
|  | Laparoscopic assistence | 80 (33.2) | 78.4 |  |
|  | Robotic basic skills | 33 (13.7) | 32.3 |  |
|  | Robotic surgery sub-steps | 29 (12) | 28.4 |  |
|  | Robotic assistence | 28 (11.6) | 27.4 |  |
|  | Other | 1 (0.4) | 1 |  |
| **Intraoperative training curriculum** | | 86 (35.7) | 100 |  |
|  | Laparoscopic surgery sub-steps | 82 (34) | 95.3 |  |
|  | Laparoscopic assistence | 79 (32.8) | 91.9 |  |
|  | Robotic surgery sub-steps | 24 (10) | 27.9 |  |
|  | Robotic assistence | 19 (7.9) | 22.1 |  |
|  | Other | 0 | 0 |  |
| **Time dedicated for training** | | 27 (11.2) |  |  |
|  | Training hours [hours/week] |  |  | 2 (1-4) |

Supplementary Material Table 3: Subcohort analysis considering only participants working at a certified MIS center
