# Supplementary material for: Satisfaction of surgeons with the current state of training in minimally invasive surgery: a survey among German surgeons
Source: Surg Endosc. 2023 Dec 12;38(2):1029–44. doi: 10.1007/s00464-023-10584-y (PMC10830590; doi:10.1007/s00464-023-10584-y)
Supplement: Supplementary file 5 — Supplementary file5 (DOCX 19 KB) [file 464_2023_10584_MOESM5_ESM.docx]

|  | | **n (%)** | **Median (25. - 75. Perc.)** |
| --- | --- | --- | --- |
| **Surgeons' satisfaction** | |  |  |
|  | ...with skills lab MIS-training situation | 1008 (100) | 50 (21-75) |
|  | ...with intraoperative MIS-training situation | 1008 (100) | 58 (30-79) |
|  | …with training equipment for MIS | 1008 (100) | 40 (11-65) |
|  | …with dedicated time for MIS-training during working hours | 1008 (100) | 20 (3-50) |

Supplementary Material Table 2: Satisfaction with training situation in MIS
